# Estimating individual health-related quality of life changes in low back pain patients

**DOI:** 10.1186/s12891-023-07093-3

**Published:** 2023-12-11

**Authors:** Ron D. Hays, Steven P. Reise, Patricia M. Herman

**Affiliations:** 1 Division of General Internal Medicine & Health Services Research, UCLA Department of Medicine, 1100 Glendon Avenue, Los Angeles, CA 90024 USA; 2 Department of Psychology, UCLA, Los Angeles, CA USA; 3 RAND Corporation, Santa Monica, CA USA

**Keywords:** Individual change, Patient-reported outcomes, PROMIS®, Estimation

## Abstract

**Background:**

There is a need to evaluate different options for estimating individual change in health-related quality of life for patients with low back pain.

**Methods:**

Secondary analysis of data collected at baseline and 6 weeks later in a randomized trial of 749 adults with low back pain receiving usual medical care (UMC) or UMC plus chiropractic care at a small hospital at a military training site or two large military medical centers. The mean age was 31; 76% were male and 67% were White. The study participants completed the Patient-Reported Outcomes Measurement Information System (PROMIS®)-29 v 1.0 physical function, pain interference, pain intensity, fatigue, sleep disturbance, depression, anxiety, satisfaction with participation in social roles, physical summary, and mental health summary scores (T-scored with mean = 50 and standard deviation (SD) = 10 in the U.S. general population).

**Results:**

Reliability estimates at the baseline ranged from 0.700 to 0.969. Six-week test–retest intraclass correlation estimates were substantially lower than these estimates: the median test–retest intraclass correlation for the two-way mixed-effects model was 0. 532. Restricting the test–retest reliability estimates to the subset who reported they were about the same as at baseline on a retrospective rating of change item increased the median test–retest reliability to 0.686. The amount of individual change that was statistically significant varied by how reliability was estimated, and which SD was used. The smallest change needed was found when internal consistency reliability and the SD at baseline were used. When these values were used, the amount of change needed to be statistically significant (*p* < .05) at the individual level ranged from 3.33 (mental health summary scale) to 12.30 (pain intensity item) T-score points.

**Conclusions:**

We recommend that in research studies estimates of the magnitude of individual change needed for statistical significance be provided for multiple reliability and standard deviation estimates. Whenever possible, patients should be classified based on whether they 1) improved significantly and perceived they got better, 2) improved significantly but did not perceive they were better, 3) did not improve significantly but felt they got better, or 4) did not improve significantly or report getting better.

## Background

Patient-reported outcome measures provide essential information about the effects of interventions on functioning and well-being [1]. The importance of supplementing group-level mean differences with estimates of responders to treatment is increasingly recognized [2]. The reliable change index (RCI) is most often used to evaluate individual change from one time point (e.g., baseline) to a follow-up [3]: individual change/($\sqrt2\;SEM)$, where SEM (standard error of measurement) is: $SD \sqrt{1-{reliability}}$, and SD is the standard deviation. However, the reliability and SD can be estimated in different ways that effect the estimated RCI and classification of whether an individual has gotten worse, stayed the same, or gotten better.

For simple-summated scales, reliability can be estimated as internal consistency reliability [4] or test–retest reliability [5]. For a measure that is a weighted combination of scale scores (i.e., a weighted composite), reliability can be estimated using Mosier’s formula [6] or test–retest reliability. Test–retest reliability can be estimated using either two-way mixed effects or random effects analysis of variance [7]. The mixed effects formula is (MS_between_ – MS_interaction_)/MS_between_, where MS_between_ is the mean square between respondents and MS_iinteraction_ is the mean square for the interaction of respondents and timepoint (test, retest). The random effects model is N (MS_between_ – MS_interaction_)/(N MS_between_, + MS_time_ – MS_interaction_), where N is the number of respondents and MS_time_ is the mean square for the main effect of the timepoint. Qin et al. [8] argued for using a "two-way mixed effect" ANOVA with interaction for absolute agreement that is equivalent to the two-way random effects model. The intraclass correlation variant of these formulas yields the estimated reliability for a single assessment.

The SD at baseline or the SD of change can be used in the RCI denominator. The choice is analogous to different denominators for responsiveness to group-level change indices [9]. The SD of change within-subjects [10] is perhaps the most consistent epistemologically with evaluating individual change. The SD of change can be estimated from the baseline and follow-up SD and the correlation between baseline and follow-up [11]:

$${SD}_{change}=\sqrt{{SD}_{baseline }^{2}{+ SD}_{followup}^{2}-(2 \times Corr \times {SD}_{baseline} \times {SD}_{followup})}$$

Significant individual change can also be estimated by using “typical error” for the standard error estimate: SD_change_/$\sqrt{2}$ [12].

In summary, multiple possible reliability and SD estimates can be used in estimating individual change. Researchers and clinicians need to understand how the choice of reliability and SD estimates impacts the classification of individual change based on the RCI. We compare different ways of estimating significant individual change for the Patient-Reported Outcomes Measurement Information System (PROMIS®)-29 v1.0 profile instrument using data from a longitudinal study of U.S. service members with low back pain [13].

## Methods

This is a secondary analysis of data collected at a small hospital in a military training site (Naval Hospital in Pensacola, Florida) and two large military medical centers in major metropolitan areas: 1) Walter Reed National Military Medical Center in Bethesda, Maryland; and 2) Naval Medical Center in San Diego, California. Study participants were randomized to usual medical care (UMC) or UMC plus chiropractic care. The active treatment period for the study was 6 weeks which served as the primary end point for the study outcomes. The clinical trial did not dictate the care to be delivered. Care was determined by the patient and their clinician. Participants in the UMC group were asked to refrain from seeking chiropractic care during the 6-week treatment period.

The PROMIS-29 v1.0 [14] was administered at baseline and 6-weeks later. It includes a single pain intensity item and 7 multi-item scales with 4 items each (physical function, pain interference, fatigue, sleep disturbance, depression, anxiety, satisfaction with participation in social roles). In addition, a pain composite (combination of pain intensity and pain interference), emotional distress composite (combination of depression and anxiety), physical health summary score, and mental health summary score can be estimated [15]. Extensive support for the reliability and validity of the PROMIS-29 profile measure has been published [14, 16, 17]. Statistically significant mean differences favoring UMC plus chiropractic care over UMC alone on all PROMIS**®**-29 v1.0 scales were previously reported [18]. All PROMIS**®**-29 v1.0 scale scores were estimated using existing calibrations (T-score metric: mean: 50, SD: 10 in U.S. general population).

A retrospective rating of change in pain was administered at the 6-week post-baseline assessment: “Compared to your first visit, your low back pain is: *much worse*, *a little worse*, *about the same*, *a little better, moderately better*, *much better*, or *completely gone*?” This item was used to identify patients who perceived that their low back had not changed during these 6 weeks.

### Analysis plan

We computed internal consistency reliability [4] for the multi-item scales, Mosier’s [6] reliability estimate for the PROMIS**®**-29 v1.0 physical and mental health summary scores, and test–retest (intraclass) correlations using analysis of variance [5]. We estimated the SD at baseline for the UMC group (SD_1_) and for the subset of the UMC group that reported they were about the same at 6 weeks compared to baseline (SD_1*_). In addition, we estimated the SD of change between baseline and 6 weeks for the UMC group (SD_2_) and the subgroup of the sample that reported at 6 weeks that they were about the same as at baseline (SD_2*_). Finally, we estimated the SD of change within subjects (SD_3_).

We estimate the magnitude of individual change between baseline and 6 weeks later needed to be significant at *p* < 0.05 using the coefficient of repeatability (CR). The CR is a re-expression of the RCI and is also known as the minimally detectable change, smallest real difference, or the smallest detectable change: CR for *p* < 0.05: 1.96 $\sqrt{2}$ SEM. We compare six different estimates of the CR: 1) CR_1_ (based on internal consistency reliability and SD_1_); 2) CR_2_ (based on internal consistency reliability and SD_1*_); 3) CR_3_ (based on random effects test–retest intraclass correlations and SD_2_); 4) CR_4_ (based on random effects test–retest intraclass correlations and SD_2*_); 5) CR_5_ (based on the SD of change within subjects) and 6) CR_6_ (based on the typical error method). Table 1 provides the six CR formulas. These CRs cover all the relevant possibilities of SDs and reliability estimates.

**Table 1:** Coefficient of repeatability formulas for *p*-value < 0.05

**Coefficient of repeatability (CR)**	**Formula**
CR_1_	2.77 SD_1_ $\sqrt{1-Alpha }$
CR_2_	2.77 SD_1*_ $\sqrt{1-Alpha }$
CR_3_	2.77 SD_2_ $\sqrt{1-ICC }$
CR_4_	2.77 SD_2*_ $\sqrt{1-ICC }$
CR_5_	2.77 SD_3_
CR_6_	1.96 SD_2*_ /$\sqrt{2}$

*Alpha* Internal consistency reliability (Cronbach [4]) or Mosier’s [6] formula for a weighted composite, *ICC* Intraclass correlation estimated from random effects analysis of variance, *SD*_*1*_ Standard deviation at baseline, *SD*_1*_ Standard deviation at baseline for subgroup reporting “about the same” on retrospective change item, *SD*_*2*_ Standard deviation of change, *SD*_2*_ Standard deviation of change for subgroup reporting “about the same” on retrospective change item, *SD*_*3*_ Standard deviation of change within subjects over two-time points

## Results

The average age of the 749 study participants was 31; 76% were male and 67% White. Most participants reported low back pain for more than 3 months (chronic low back pain, 51%), 38% had acute and 11% had subacute low back pain.

Internal consistency and weighted composite reliability estimates ranged from 0.700 to 0.969 (Table 2). Six-week test–retest intraclass correlation estimates were substantially lower than these estimates. The median test–retest reliability estimate for the two-way mixed effects model was 0.532 and ranged from 0.359 (pain composite) to 0.647 (emotional distress composite) in the UMC group overall. The estimated median test–retest reliability was 0.686 and reliabilities ranged from 0.550 (satisfaction with participation in social roles) to 0.765 (physical health summary) within the subset of the sample who reported they were *about the same* compared to baseline on the retrospective rating of change item. The test–retest reliability estimates based on the random effects model were similar but tended to be a little lower than those based on the mixed effects model.

**Table 2:** Reliability of PROMIS-29 v. 1.0 scales

Scale	Internal Consistency Reliability in Overall Sample (*n* = 749)	ICC for Usual Medical Care Group	ICC for stable subgroup in Usual Medical Care Group
Physical function	0.898	0.482 (.451)	0.745 (.731)
Pain interference	0.936	0.362 (.331)	0.640 (.614)
Pain intensity	0.681^a^	0.400 (.382)	0.679 (.681)
Fatigue	0.935	0.619 (.612)	0.620 (.619)
Sleep Disturbance	0.820	0.620 (.609)	0.709 (.706)
Depression	0.891	0.649 (.650)	0.684 (.673)
Anxiety	0.880	0.582 (.575)	0.626 (.628)
Satisfaction with participation in social roles	0.964	0.420 (.415)	0.550 (.552)
Pain composite	0.874^b^	0.359 (.331)	0.687 (.680)
Emotional distress composite	0.930^b^	0.647 (.646)	0.704 (.704)
Physical health summary	0.924^b^	0.483 (.452)	0.765 (.753)
Mental Health summary	0.969^b^	0.619 (.603)	0.741 (.742)

*ICC* Intraclass correlation from two-way mixed effects model (random effects model estimates in parentheses)

^a^Estimated for single item based on ICC

^b^Mosier’s [6] formula used to estimate reliability

Table 3 provides the SD and CR estimates. The smallest SDs were found for the standard deviation of change within the subgroup that reported they didn’t change from baseline to 6 weeks later (SD_3_). The smallest CRs tended to be those derived from SD_1_ in combination with internal consistency reliability estimates (CR_1_). These smallest CRs ranged from 3.33 (mental health summary scale) to 12.30 (pain intensity item).

**Table 3:** Coefficient of Repeatability (CR) using different reliability and standard deviation estimates

PROMIS-29 Scale	SD_1_ (*n* = 316)	SD_1*_ (*n* = 119)	SD_2_ (*n* = 316)	SD_2*_ (*n* = 119)	SD_3_	CR_1_	CR_2_	CR_3_	CR_4_	CR_5_	CR_6_
Physical function	7.12	7.19	7.85	5.36	3.92	6.30	6.36	11.28	7.70	10.87	7.43
Pain interference	7.32	7.25	9.03	6.35	4.74	5.13	5.08	15.54	10.93	13.13	8.80
Pain intensity	7.86	8.63	9.59	6.59	4.64	12.30	13.50	15.00	10.31	12.86	9.13
Fatigue	9.53	8.76	9.14	8.57	6.06	6.73	6.19	15.63	14.65	16.79	11.88
Sleep disturbance	7.43	7.07	7.03	5.90	4.21	8.73	8.31	10.56	8.86	11.65	8.18
Depression	6.72	6.42	6.00	5.61	4.07	6.15	5.87	9.50	8.89	11.28	7.78
Anxiety	8.69	8.60	8.23	7.61	5.36	8.34	8.25	12.90	12.86	14.86	10.55
Satisfaction with participation in social roles	8.83	8.04	9.97	7.92	5.58	4.64	4.23	18.48	14.68	15.45	10.98
Pain composite	6.61	6.85	8.50	5.39	3.88	6.50	6.74	13.32	8.45	10.75	7.47
Emotional distress	6.95	6.89	6.28	5.73	4.06	5.09	5.05	9.46	8.64	11.24	7.94
Physical health summary	7.30	7.25	8.12	5.23	3.82	5.57	5.54	11.18	7.20	10.57	7.25
Mental health summary	6.82	6.29	6.72	4.93	3.48	3.33	5.07	9.45	6.94	9.65	6.83

All scale scores were estimated using existing calibrations (T-score metric: mean = 50, SD = 10 in U.S. general population)

SD_1_ SD baseline for the usual medical care group, SD_1*_ SD baseline for subgroup of usual medical care group reporting “about the same” on retrospective change item, SD_2_ SD change for usual medical care group, SD_2*_ SD change for subgroup of usual medical care group reporting “about the same”, SD_3_ SD within (MS_error_ * 2.77) for subgroup reporting “about the same”, CR_1_ coefficient of repeatability (CR) based on internal consistency reliability and SD_1_, CR_2_ coefficient of repeatability (CR) based on internal consistency reliability and SD_1*_, CR_3_ CR based on random effects test–retest intraclass correlation and SD_2_, CR_4_ CR based on random effects test–retest intraclass correlation and SD_2*_, CR_5_ CR based on SD_3_, CR_6_ CR based on 1.96 * SD_2*_ /$\sqrt{2 }$

## Discussion

This study shows varying estimates of the CR when using different ways of estimating reliability and the SD. The smallest CR was obtained when internal consistency reliability and the SD at baseline for the UMC sample were used. The different SDs used to evaluate individual change are analogous to options for estimating responsiveness of measures to group-level change [19]. Responsiveness indices include group mean change in the numerator and the same SDs examined in this study for the denominator: effect size uses SD_1_, the standardized response mean uses SD_2,_ and the responsiveness statistic uses SD_2*_. These results provide concrete information that the way that the RCI and CR are estimated impacts whether an individual is deemed to have stayed the same or changed over time on patient-reported outcome measures.

While some have suggested that test–retest reliability and the SD of change provide the cleanest estimates for use in evaluating within change from baseline to follow-up, there are practical challenges in using them. Reeve et al. [20]:

“noted practical concerns regarding test–retest reliability, primarily that some populations studied in PCOR are not stable and that their HRQOL can fluctuate. This phenomenon would reduce estimates of test–retest reliability, making the PRO measure look unreliable when it may be accurately detecting changes over time. In addition, memory effects will positively influence the test–retest reliability when the two survey points are scheduled close to each other.”

But the impact of different reliability and SD estimates on the CR depends on the context. Test–retest reliability estimates were all below the 0.90 threshold for use of measures to assess individuals [7]. These were likely underestimates of reliability because of the 6-week interval between assessments in a sample of individuals with chronic back pain. Future studies are needed that use shorter intervals of time for test–retest estimates. Caution is warranted in generalizing from a sample of active-duty members of the U.S. military. Further comparison of the SD alternatives is needed in other samples and with different measures. It also may be informative to assess the same issues with different individual change indices such as the standard error of prediction (SEP), which uses the (${SD}_{1} \sqrt{1-{reliability}^{2}}$) in the denominator [21]. In addition, future studies should consider using item response theory standard error estimates rather than one reliability estimate applied to every individual [22].

Significant individual change is conceptually different from group-level estimates of the minimally important change (MIC) for patient-reported outcome measures. Classifying individuals as changed using MIC estimates is inappropriate and results in overly optimistic estimates of responders to treatment [2]. However, concerns about the seemingly large amount of individual change needed to be significant at *p* < 0.05 have been raised [23, 24]. Lower levels of confidence may be appropriate to monitor short-term change when a trend is expected to continue over time [25]. Donaldson [23] suggested that a less stringent confidence interval than 95% could be used to classify people as likely having changed or staying the same on a patient-reported outcome measure. Doing this results in a smaller CR and a test of significance that is more sensitive but less specific to perceived change by patients. In this study CR_1_ was smaller than CR_2_ (Table 3). Sensitivity to retrospectively reported improvement in low back pain (*a little better*, *moderately better*, *much better*, or *completely gone*) was higher and specificity lower for CR_1_ than CR_2_. For example, with the physical function scale the sensitivity of CR_1_ to retrospective reports of improvement was 46% compared to 29% for CR_2_ but the specificity of CR_1_ to reported improvement was 85% versus 98% for CR_2_.

In addition to whether change is statistically significant, where the individual is at follow-up may be important in clinical practice. That is, the focus could be on bringing the patient to the normal range of a clinical parameter. For example, a clinician might focus on whether their therapy takes someone who starts with hypertension to within the normal range. Similarly, for patient-reported outcomes, a clinician might be interested in whether the patient who is clinically depressed at baseline is no longer depressed at follow-up.

## Conclusions

We recommend that the sensitivity of results be evaluated for different reliability and SD estimates in research studies evaluating individual change. For assessing whether individuals have changed in clinical practice, we suggest clinicians estimate significant individual change for simple summated scales using CR_1_ (internal consistency reliability and the SD at baseline). If possible, they should also ask individuals at follow-up if they have changed. Having information about significant individual change on the patient-reported outcome measure and the individual’s perception of whether they have changed, the clinician can classify an individual patient as: 1) improved significantly and perceived they got better (i.e., reported their low back pain was a little better, moderately better, much better, or completely gone), 2) improved significantly but did not perceive they were better (i.e., reported their low back pain was about the same, a little worse, or much worse), 3) did not improve significantly but perceived they got better, and 4) did not improve significantly and did not perceive they were better.

## Data Availability

This secondary analysis uses an existing dataset. The study was registered at ClinicalTrials.gov (NCT01692275). Requests for the data can be directed to: Patricia Herman, ND, PhD, RAND Corporation, 1776 Main Street, Santa Monica, CA 90401 (pherman@rand.org).

## Abbreviations

Alpha: Internal consistency reliability
CR: Coefficient of repeatability
CR_1_: Coefficient of repeatability (CR) based on internal consistency reliability and SD_1_
CR_2_: Coefficient of repeatability (CR) based on internal consistency reliability and SD_1*_
CR_3_: CR based on random effects test–retest intraclass correlation and SD_2_
CR_4_: CR based on random effects test–retest intraclass correlation and SD_2*_
CR_5_: CR based on SD_3_
CR_6_: CR based on 1.96 * SD_2*_ /$\sqrt{2 }$
ICC: Intraclass correlation
MS_between_: Mean square between respondents
MS_nteraction_: Mean square for the interaction of respondents and timepoint (test, retest)
MS_time_: Mean square for main effect of timepoint
N: The number of respondents
PROMIS: Patient-Reported Outcomes Measurement Information System
RCI: Reliable change index
SD: Standard deviation
SD_1_: Standard deviation at baseline for the usual medical care group
SD_1*_: SD baseline for subgroup of the usual medical care group reporting “about the same” on retrospective change item
SD_2_: Standard deviation of change for the usual medical care group
SD_2*_: Standard deviation of change for subgroup of the usual medical care group reporting “about the same” on retrospective change item
SD_3_: Standard deviation of change within subjects for subgroup of the usual medical care group reporting “about the same” on retrospective change item
SEM: Standard error of measurement
UMC: Usual medical care
